# Clinical dosage of meclozine promotes longitudinal bone growth, bone volume, and trabecular bone quality in transgenic mice with achondroplasia

**DOI:** 10.1038/s41598-017-07044-8

**Published:** 2017-08-07

**Authors:** Masaki Matsushita, Ryusaku Esaki, Kenichi Mishima, Naoki Ishiguro, Kinji Ohno, Hiroshi Kitoh

**Affiliations:** 1 Department of Orthopaedic Surgery, Nagoya University Graduate School of Medicine, 65 Tsurumai, Showa-ku, Nagoya 466-8550 Japan; 2 Division of Neurogenetics, Center for Neurological Diseases and Cancer, Nagoya University Graduate School of Medicine, 65 Tsurumai, Showa-ku, Nagoya 466-8550 Japan

## Abstract

Achondroplasia (ACH) is the most common short-limbed skeletal dysplasia caused by gain-of-function mutations in the fibroblast growth factor receptor 3 (FGFR3). No effective FGFR3-targeted therapies for ACH are currently available. By drug repositioning strategies, we identified that meclozine, which has been used as an anti-motion-sickness, suppressed FGFR3 signaling in chondrocytes and rescued short-limbed phenotype in ACH mouse model. Here, we conducted various pharmacological tests for future clinical application in ACH. Pharmacokinetic analyses demonstrated that peak drug concentration (C_max_) and area under the concentration-time curve (AUC) of 2 mg/kg of meclozine to mice was lower than that of 25 mg/body to human, which is a clinical usage for anti-motion-sickness. Pharmacokinetic simulation studies showed that repeated dose of 2 mg/kg of meclozine showed no accumulation effects. Short stature phenotype in the transgenic mice was significantly rescued by twice-daily oral administration of 2 mg/kg/day of meclozine. In addition to stimulation of longitudinal bone growth, bone volume and metaphyseal trabecular bone quality were improved by meclozine treatment. We confirmed a preclinical proof of concept for applying meclozine for the treatment of short stature in ACH, although toxicity and adverse events associated with long-term administration of this drug should be examined.

## Introduction

Achondroplasia (ACH) is one of the most common skeletal dysplasias characterized by severe short stature with rhizomelic shortening of the extremities, relative macrocephaly with frontal bossing, midface hypoplasia, and increased lumbar lordosis^1^. ACH is caused by activating mutations in *FGFR3* encoding the fibroblast growth factor receptor 3, which is a negative regulator of longitudinal bone growth^2, 3^. Foramen magnum stenosis, hydrocephalus, and spinal canal stenosis are potentially serious complications of ACH^4^. In addition, osteoporotic features were also observed in patients with ACH^5^ as well as in mouse model of ACH^6^. Histologically, FGFR3 is expressed in proliferative and prehypertrophic zones of the growth plate and modulates both proliferation and differentiation of the chondrocytes^7^.

No radical treatments for short stature in ACH are currently available. Recombinant human growth hormone has been administered for short stature in ACH children in some limited countries^8^, but the response to this therapy is moderate and the long-term effects remain controversial. Distraction osteogenesis is another therapeutic option to gain longitudinal bone length but it needs significant time and efforts associated with higher rates of complications^9^. Several FGFR3-targeted treatments for ACH have experimentally been applied in recent years. Yamashita *et al*.^10^ demonstrated that statins rescued the dwarf phenotype of ACH mouse model by using induced pluripotent stem (iPS) cells established from patients with gain-of-function mutations in *FGFR3*. A specific inhibitor for FGFR3, NVP-BGJ398, not only increased longitudinal bone growth but also ameliorated the foramen magnum stenosis in mouse model of ACH^11^. Continuous intravenous administration of C-type natriuretic peptide (CNP) promoted longitudinal bone growth^12^ and the CNP analog, BMN-111, showed significant bone growth recovery by daily subcutaneous administration in mutant mice^13^. Furthermore, BMN-111 is undergoing a human clinical trial (ClinicalTrials.gov NCT02055157).

Drug repositioning, which is the application of approved drugs for new indications, has been reported cost- and time-effective strategies to identify a therapy for at least a subset of orphan diseases^14^. By comprehensive drug screening, we identified that meclozine, an anti-histamine drug that has been used as an anti-motion-sickness for more than 50 years, inhibited FGFR3 signaling in various chondrocytic cell lines^15^. In addition, oral administration of *ad libitum* intake of meclozine-containing foods increased longitudinal bone growth in ACH mouse model^16^. The pharmaceutical activity of meclozine mixed by the foods, however, is likely to be decreased as the storage period gets longer. Moreover, accurate drug dosage cannot be measured by *ad libitum* administration. In the current study, we performed pharmacokinetic and pharmacological studies of meclozine and determined the optimal effective dose of the drug on promoting longitudinal bone growth using the ACH mouse model. We further demonstrated additional FGFR3 inhibitory effects of this drug on bone volume and bone quality.

## Results

### Administration of 2 mg/kg of meclozine to mice is clinically relevant

**Figure 1:**
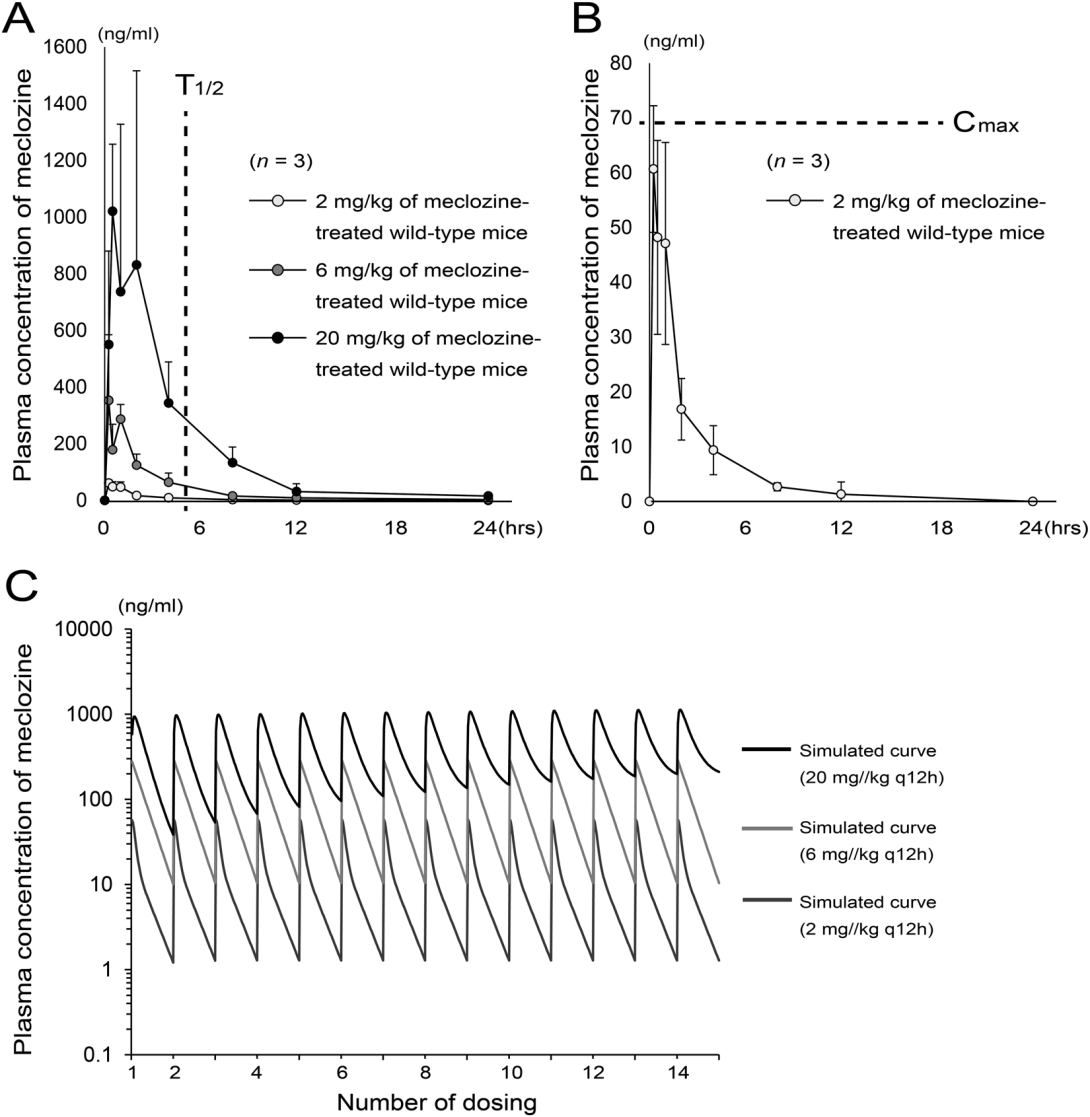
Pharmacokinetics after administration of 2 mg/kg of meclozine to mice is clinically relevant. (**A**) Plasma concentration-time course of meclozine was shown in 8-week-old wild-type mice after single administration of 2, 6, or 20 mg/kg of meclozine. The mean terminal elimination half-life (T_1/2_) after a single dose of 25 mg of meclozine in human subjects was 5.21 hours (dotted line)^17^. Elevated plasma concentrations of meclozine were observed in a dose dependent manner (*n* = 3 for each group and each time point). (**B**) The average plasma concentrations of meclozine after 2 mg/kg administration were within the mean peak drug concentration (C_max_) after a single dose of 25 mg of meclozine in human subjects (dotted line)^17^. Mean and SD are indicated. (**C**) There are no accumulation effects by repeated administration of 2 mg/kg of meclozine. Simulated curves of plasma concentration-time course of meclozine during repeated administration of 2, 6, or 20 mg/kg every 12 hours for seven days. Cumulative pharmacokinetics, which was observed in repeated administration of 20 mg/kg of meclozine, was not seen by repeated administration of 2 or 6 mg/kg of meclozine. ‘q 12 h’ means every 12 hours.

Pharmacokinetics of meclozine after administration of 2, 6, or 20 mg/kg was demonstrated in Table 1. The peak drug concentration (C_max_) and time to peak drug concentration (T_max_) of 2, 6, and 20 mg/kg of meclozine were 60.7, 352.4, and 1020.0 ng/ml, and 0.25, 0.25, and 0.50 hours, respectively. Dose dependent elevation of plasma concentration of meclozine was observed (Fig. 1A). The half-life of 2, 6, or 20 mg/kg of meclozine was 2.66, 6.43, and 3.82 hours, respectively. The C_max_ after administration of 25 mg/body to human (68.42 ng/ml) was larger than that after administration of 2 mg/kg to mice (Fig. 1B). Moreover, the area under the concentration-time curve from time zero to the last sampling time 24 hours (AUC_0-24_) and area under the concentration-time curve from time zero to infinity (AUC_inf_) after administration of 25 mg/body to human were 446.52 ng·hour/ml and 464.53 ng·hour/ml, respectively^17^, which were larger than those after administration of 2 mg/kg of meclozine in the current study.

**Table 1:** Pharmacokinetic parameters after meclozine administration.

	**Human** ^17^	**Mice**		
	**25 mg/body**	**2 mg/kg**	**6 mg/kg**	**20 mg/kg**
C_max_ (ng/mL)	68.4	60.7	352.4	1020.0
T_max_ (h)	3.11	0.25	0.25	0.50
T_1/2_ (h)	5.11	2.66	6.43	3.82
AUC_0-24h_ (ng·h/mL)	446.5	143.2	900.5	4223.0
AUC_inf_ (ng·h/mL)	464.5	140.4	925.5	4308.4

C_max_ = Peak drug concentration; T_max_ = Time to peak drug concentration; T_1/2_ = Terminal elimination half-life; AUC_0–24h_ = Area under the concentration-time curve from time zero to the last sampling time; AUC_inf_ = Area under the concentration-time curve from zero to infinity.

We next demonstrated the simulated curve of plasma concentration of meclozine which was administered at intervals of 12 hours for 7 days at various concentrations (2, 6, and 20 mg/kg). Cumulative pharmacokinetics was observed in repeated administration of 20 mg/kg of meclozine, while there was no cumulation of the substance found in 2 mg/kg and 6 mg/kg of dosage (Fig. 1C). Similarly, pharmacokinetics after the first and last (14th dose) administration demonstrated completely overlapped curves in 2 mg/kg and 6 mg/kg of dosage, and elevated plasma concentrations after the last administration in 20 mg/kg (Supplementary Fig. S1A–C).

### Short stature phenotype of *Fgfr3*^ach^ mice was rescued by twice-daily administration of 1 and 2 mg/kg/day of meclozine for 10 days

**Figure 2:**
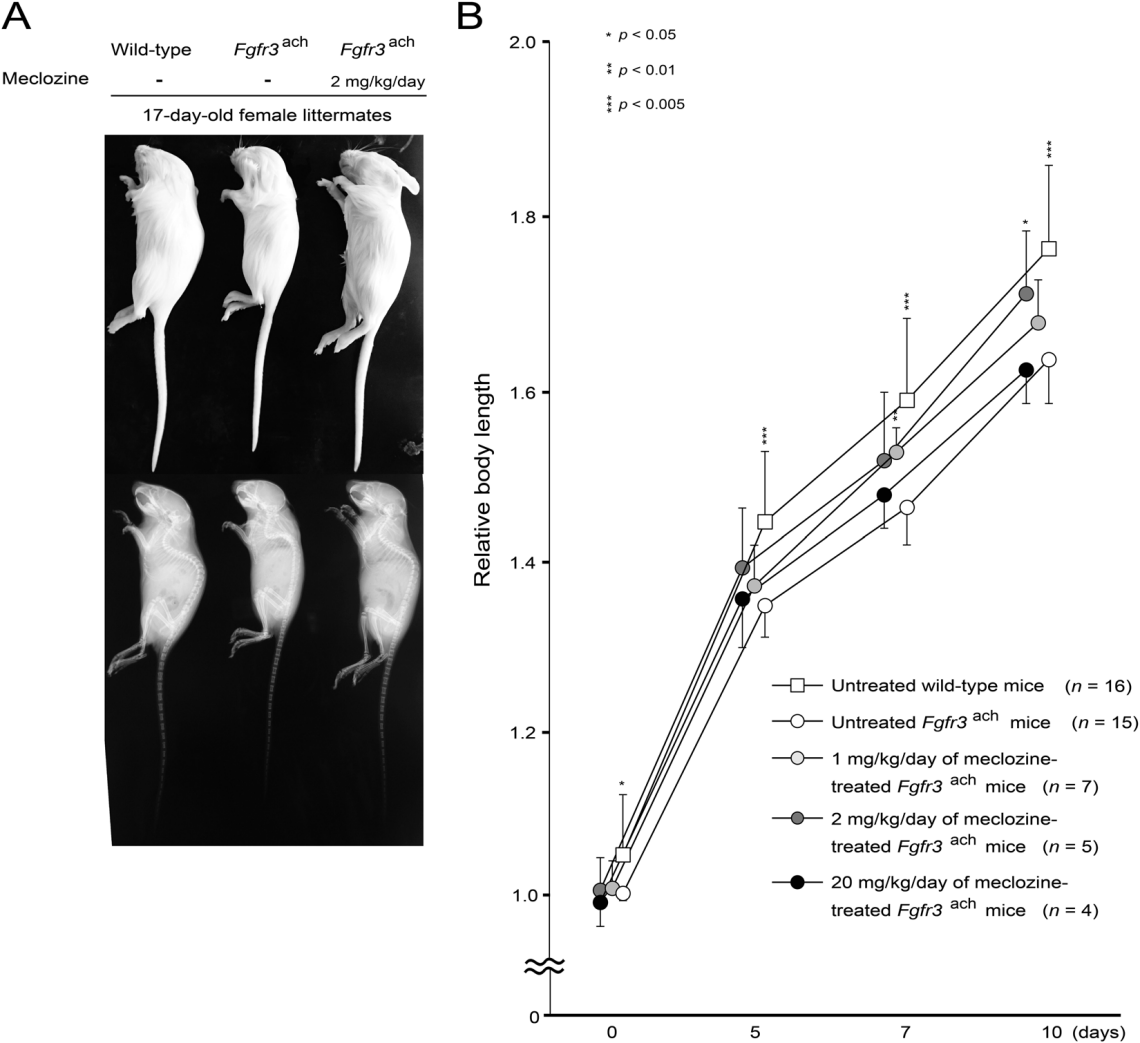
Twice-daily oral administration of 1 and 2 mg/kg/day of meclozine reverses the dwarfed phenotype in *Fgfr3* ^ach^ mice. (**A**) A representative image of the individual female littermates at the end of treatment demonstrated that an untreated *Fgfr3* ^ach^ mouse reveled the dwarfed phenotype, which was rescued by oral administration of 2 mg/kg/day of meclozine treatment. (**B**) Relative body length of the wild-type mice and *Fgfr3* ^ach^ mice was calculated based on the body length of untreated *Fgfr3* ^ach^ mice at 0 day. The body length of 1 or 2 mg/kg/day of meclozine-treated *Fgfr3* ^ach^ mice was larger than that of untreated *Fgfr3* ^ach^ mice throughout the treatment period. On the other hand, 20 mg/kg/day of meclozine-treated *Fgfr3* ^ach^ mice were not larger than untreated *Fgfr3* ^ach^ mice at the end of treatment. Mean and SD are indicated. Statistical significance was analyzed by the unpaired *t* test for each dose of meclozine-treated *Fgfr3* ^ach^ mice (*n* = 7, 5, and 4 for 1, 2, and 20 mg/kg/day of meclozine, respectively) or untreated wild-type mice (*n* = 16) versus untreated *Fgfr3* ^ach^ mice (*n* = 15).

We measured body length at 0, 5, 7, and 10 days after administration of meclozine. At the end of meclozine treatment twice a day for 10 days, the body length of the untreated *Fgfr3* ^ach^ mice was shorter than that of the sex-matched wild-type littermates. In contrast, the body length of the 2 mg/kg/day of meclozine-treated *Fgfr3* ^ach^ mice was closer to that of sex-matched wild-type littermates (Fig. 2A). Temporal analyses revealed that the body lengths of wild-type mice were larger than those of untreated *Fgfr3* ^ach^ mice through the experimental periods. After meclozine treatment for 10 days, the body lengths of 1 and 2 mg/kg/day of meclozine-treated *Fgfr3* ^ach^ mice were increased by 3.0% (*p* = 0.69) and 6.7% (*p* < 0.05), respectively. The 20 mg/kg/day of meclozine did not show any growth promotion effects (Fig. 2B and Supplementary Figs S2A and S3A). On the other hand, once-daily administration of meclozine did not increase the body length of *Fgfr3* ^ach^ mice at all doses (Supplementary Fig. S4). Therefore, following examinations were performed for mice treated with twice-daily administration.

### The 1 and 2 mg/kg/day of meclozine enhanced the bone growth of *Fgfr3*^ach^ mice

**Figure 3:**
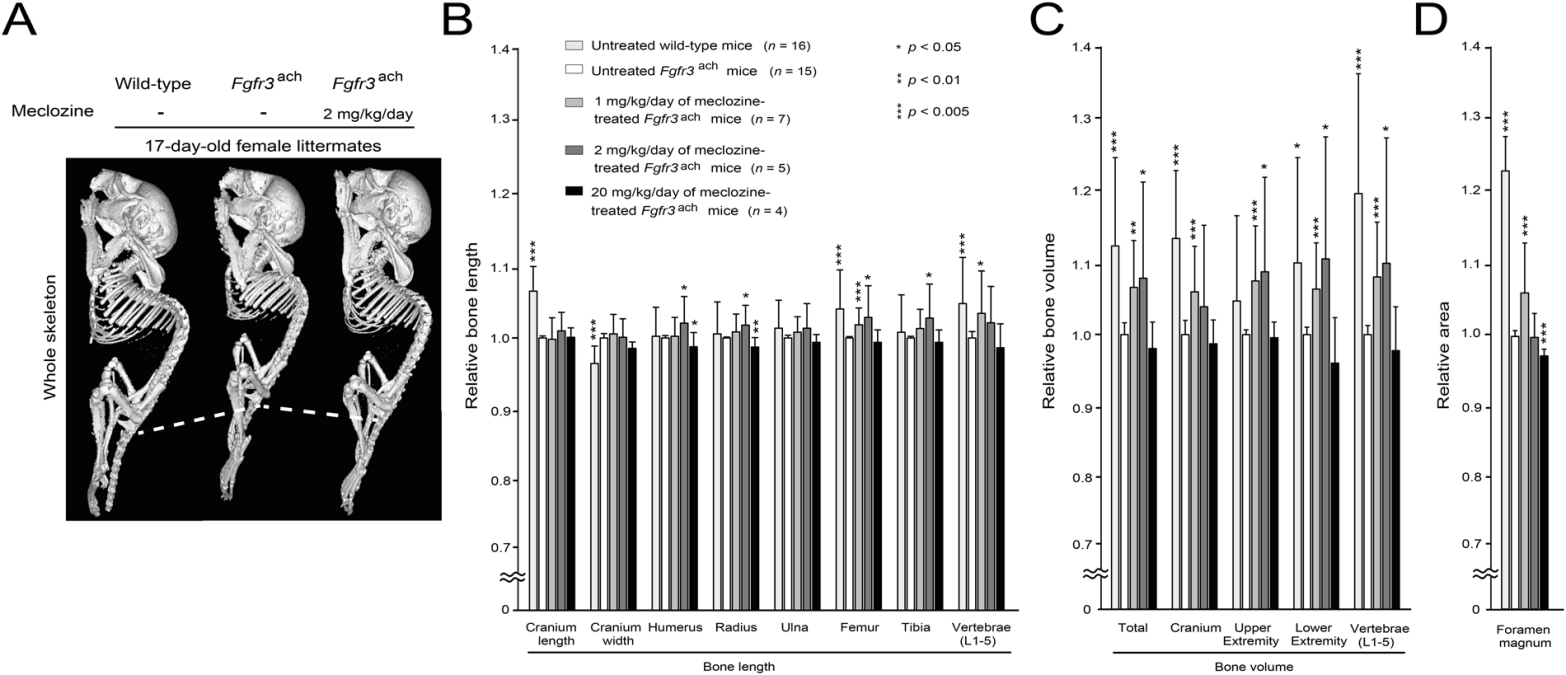
Quantitative 3D-CT examinations reveal increased bone length and bone volume of long tubular bones and vertebrae in meclozine-treated *Fgfr3* ^ach^ mice. (**A**) A representative reconstructed micro-CT image of the individual female littermates at the end of treatment showed that the body length was increased by twice-daily administration of 2 mg/kg/day of meclozine. Dotted lines indicate the inferior margin of the ischium. (**B**) Relative bone lengths of long tubular bones and vertebrae were increased after twice-daily administration of 1 or 2 mg/kg/day of meclozine. (**C**) Relative bone volumes of the cranium, upper and lower extremities, and vertebrae were significantly increased after meclozine treatment. (**D**) Only 1 mg/kg/day of meclozine enhanced the areas of foramen magnum, which did not chang by 2 mg/kg/day of meclozine treatment. Relative length, volume, and area were calculated based on those of untreated *Fgfr3* ^ach^ mice. Mean and SD are indicated. Statistical significance was analyzed by the unpaired *t* test for each dose of meclozine-treated *Fgfr3* ^ach^ mice (*n* = 7, 5, and 4 for 1, 2, and 20 mg/kg/day of meclozine, respectively) or untreated wild-type mice (*n* = 18) versus untreated *Fgfr3* ^ach^ mice (*n* = 15).

We next quantified the effect of 1, 2, and 20 mg/kg/day of meclozine on longitudinal bone growth of *Fgfr3* ^ach^ mice by three dimensional-computed tomography (3D-CT) analyses. The total bone volumes of 1 and 2 mg/kg/day of meclozine-treated *Fgfr3* ^ach^ mice were 6.9% (*p* < 0.01) and 8.2% (*p* < 0.05) larger than that of untreated *Fgfr3* ^ach^ mice, respectively (Fig. 3A and Supplementary Figs S2B and S3B). The lengths of long tubular bones and vertebrae were increased by 1 or 2 mg/kg/day of meclozine treatment in *Fgfr3* ^ach^ mice (Fig. 3B). Relative bone volumes of cranium, upper extremity, lower extremity, and vertebrae, were significantly increased in 1 or 2 mg/kg/day of meclozine-treated *Fgfr3* ^ach^ mice than those in untreated *Fgfr3* ^ach^ mice (Fig. 3C). The area of foramen magnum was increased by 1 mg/kg/day of meclozine treatment, but it did not change by 2 mg/kg/day of meclozine treatment (Fig. 3D).

### Metaphyseal trabecular bone formation was enhanced by meclozine treatment in *Fgfr3*^ach^ mice

**Figure 4:**
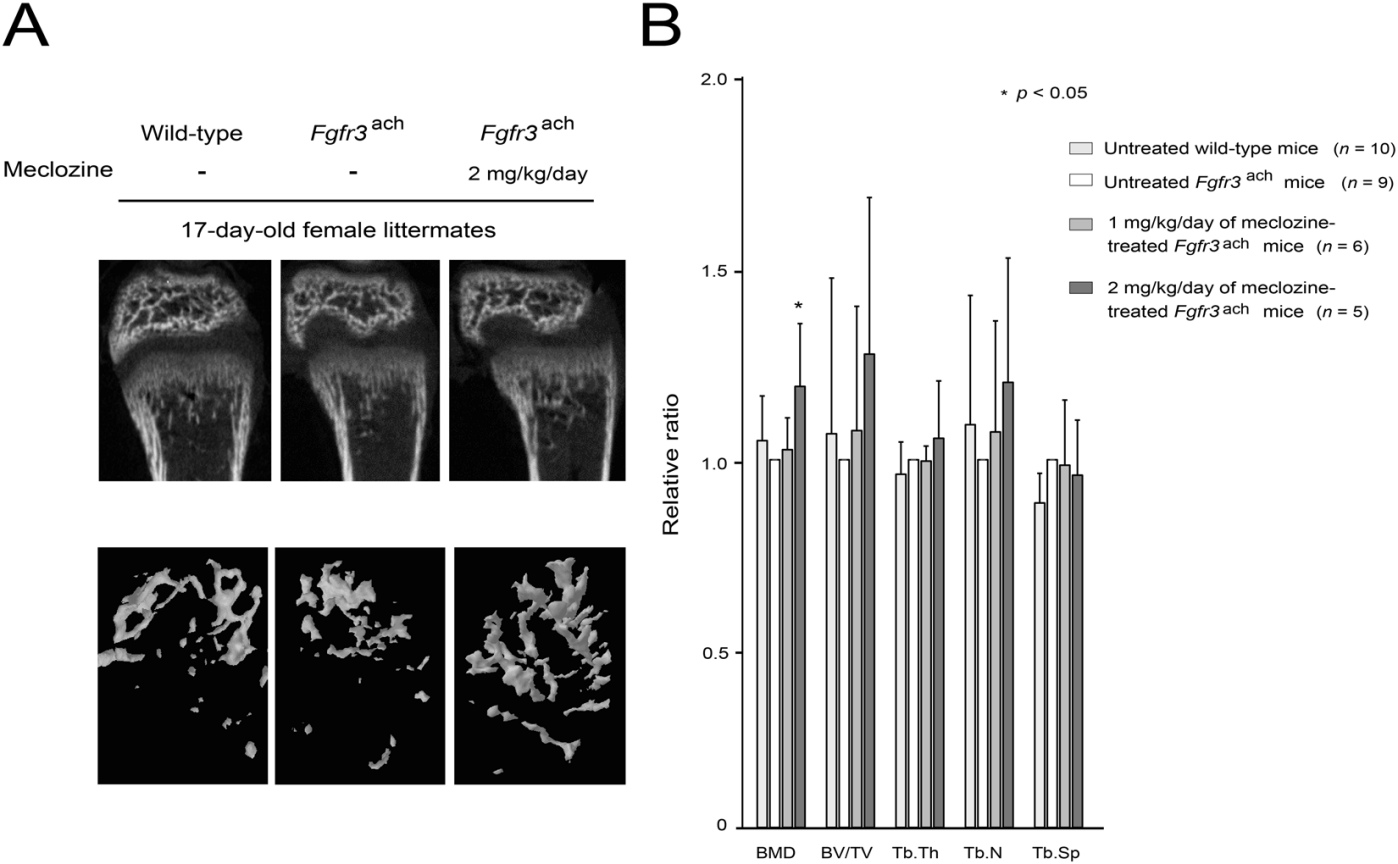
Bone mineral density is enhanced in meclozine-treated *Fgfr3* ^ach^ mice. (**A**) Representative reconstructed micro-CT images of the distal femur at the end of treatment revealed that metaphyseal bone mineral density was increased after meclozine treatment. Upper panels show 2D images of the distal femur and lower panels show 3D images of metaphyseal trabecular bone. (**B**) The average relative ratio of bone mineral density (BMD) was increased after meclozine treatment while there were no statistical differences in bone volume/total volume (BV/TV), trabecular thickness (Tb.Th), trabecular number (Tb.N), and trabecular separation (Tb.Sp). Relative values were calculated based on those of untreated *Fgfr3* ^ach^ mice. Mean and SD are indicated. Statistical significance was analyzed by the unpaired *t* test for each dose of meclozine-treated *Fgfr3* ^ach^ mice (*n* = 6 and 5 for 1 and 2 mg/kg/day of meclozine, respectively) or untreated wild-type mice (*n* = 10) versus untreated *Fgfr3* ^ach^ mice (*n* = 9).

Decreased bone mass has been reported in gain-of-function mutation of FGFR3^6^. We next evaluated the effect of 1 or 2 mg/kg/day of meclozine on the trabecular bone formation of distal femoral metaphysis in *Fgfr3* ^ach^ mice using micro-CT scans. The bone mineral density (BMD) was increased in 2 mg/kg/day of meclozine-treated *Fgfr3* ^ach^ mice by 19.9% (*p* < 0.05) than that in untreated *Fgfr3* ^ach^ mice (Fig. 4A). The trabecular thickness (Tb.Th) tended to be increased by meclozine treatment while the effects of meclozine on the bone volume/total volume (BV/TV), trabecular number (Tb.N), and trabecular separation (Tb.Sp) were not concluded (Fig. 4B).

**Figure 5:**
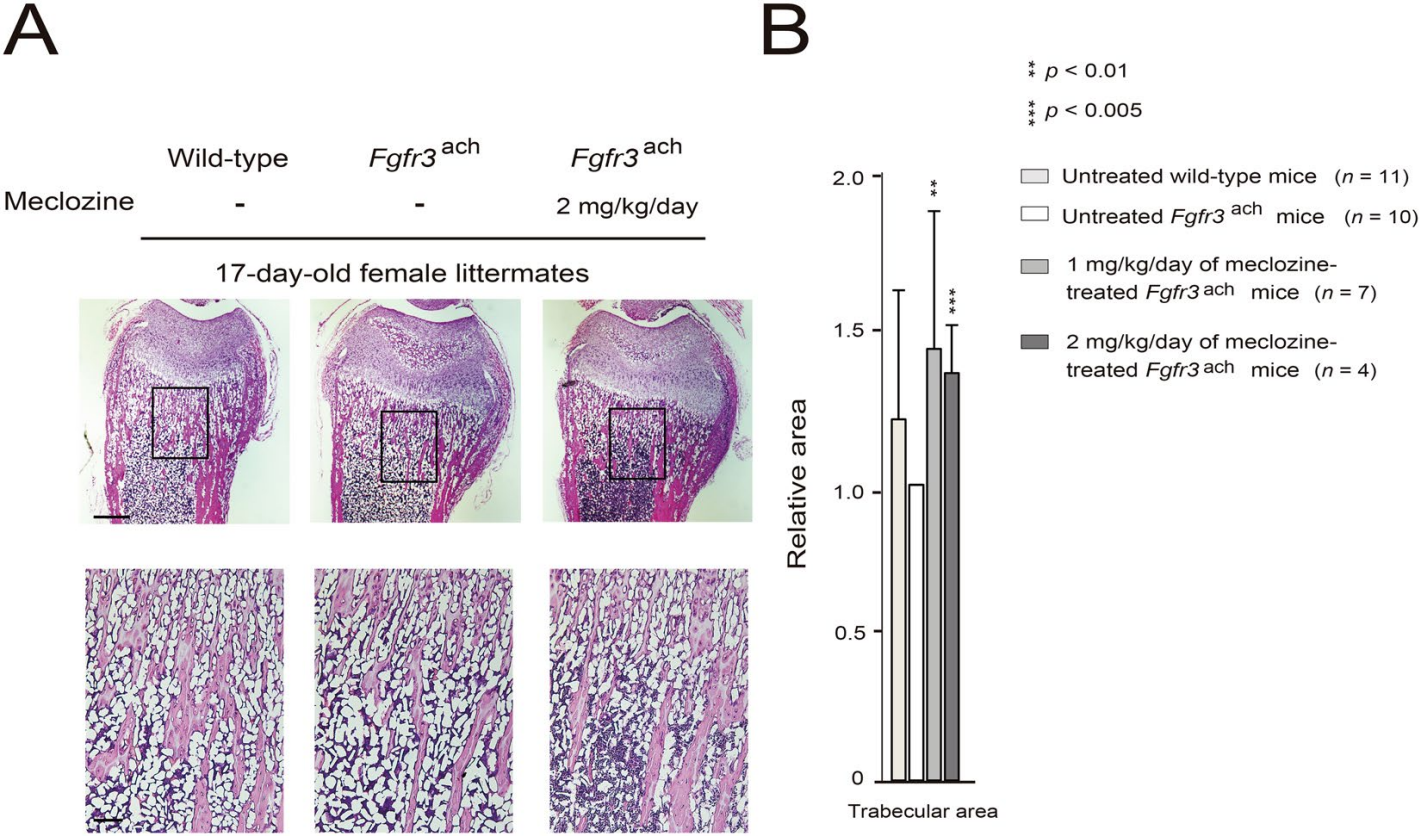
Administration of meclozine increases the trabecular bone area in the metaphysis of *Fgfr3* ^ach^ mice. (**A**) Representative decalcified histology images of the distal femur stained with hematoxylin-eosin (HE) demonstrated that the trabecular bone was more abundant in 2 mg/kg/day of meclozine-treated *Fgfr3* ^ach^ mouse than in untreated *Fgfr3* ^ach^ mouse. Squared parts are magnified in lower panels. Scale bares indicate 400 μm in upper panels and 100 μm in lower panels. (**B**) Quantitative analyses of the trabecular area stained by HE showed that relative trabecular area was significantly increased by administration of 1 or 2 mg/kg/day of meclozine in *Fgfr3* ^ach^ mice. Relative areas of the trabecular bones were calculated based on those of untreated *Fgfr3* ^ach^ mice. Mean and SD are indicated. Statistical significance was analyzed by the unpaired *t* test for each dose of meclozine-treated *Fgfr3* ^ach^ mice (*n* = 7 and 4 for 1 and 2 mg/kg/day of meclozine, respectively) or untreated wild-type mice (*n* = 11) versus untreated *Fgfr3* ^ach^ mice (*n* = 10).

We next analyzed the trabecular bone formation histologically. The trabecular bone formation in *Fgfr3* ^ach^ mice was enhanced by 1 and 2 mg/kg/day of meclozine treatment (Fig. 5A and Supplementary Fig. S5). Quantitative measurements demonstrated that the trabecular area was 45.8% (*p* < 0.01) and 37.6% (*p* < 0.005) larger in 1 and 2 mg/kg/day of meclozine-treated *Fgfr3* ^ach^ mice than in untreated *Fgfr3* ^ach^ mice, respectively (Fig. 5B).

## Discussion

Meclozine has long been used for anti-motion-sickness as a medical drug or an over-the-counter drug in many countries. It has been applied not only for adult but also for children. Safety in single dose, therefore, has already been established. For repositioning of meclozine to the treatment of short stature in ACH, we should determine the optimal dose and usage for longitudinal growth stimulation and verify safety in repeated administration of this drug. We first demonstrated that C_max_ and AUC after single dose of 2 mg/kg of meclozine in mice was lower than those after single dose of 25 mg tablet used in human^17^, and confirmed that this dose is clinically relevant. Half-life of this drug was shorter in mice than in human. We next showed that twice-daily administration of 1or 2 mg/kg/day of meclozine was more effective for bone growth of *Fgfr3* ^ach^ mice than once daily administration. No growth promoting effect of once-daily administration of meclozine seemed to be due to its short half-life. Maintaining the plasma concentration of meclozine may be important for accelerating bone growth. In regard to toxicity related to repeated administration, cumulative pharmacokinetics was never observed in twice-daily administration of 2 or 6 mg/kg of meclozine. On the other hand, an accumulation effect of 20 mg/kg of meclozine was observed, which may be related to ineffectiveness of bone growth by its toxicity. Although further studies are needed to examine toxicity and adverse events associated with long-term administration of this drug for future clinical application, we could confirm a minimum preclinical proof of concept for applying meclozine for the treatment of short stature in ACH.

Bone volume relative to bone length was markedly reduced in *Fgfr3* ^ach^ mice compared to wild-type mice in the current study, suggesting that gain-of-function mutation in FGFR3 decreases bone width as well as longitudinal bone length. Twigg *et al*.^18^ demonstrated decreased cortical bone thickness of the long bones in *Fgfr3* ^*P244R*^ mice exhibiting a ligand-dependent activating mutation in FGFR3. Significant increase in bone volume after meclozine treatment in mutant mice showed that meclozine increased bone length and bone width by suppressing FGFR3 signaling in *in-vivo*. Using *Fgfr3* ^Y367C/+^ mice exhibiting a gain-of-function mutation in FGFR3, Di Rocco *et al*.^19^ demonstrated bone defects within the frontal bones and suggested that gain-of-function in FGFR3 inhibited not only endochondral ossification but also membranous ossification. In the current study, treatment of 1 or 2 mg/kg/day of meclozine significantly increased cranium bone volume without affecting cranium length and width. This observation suggested that meclozine increased cranium bone thickness by stimulating membranous ossification.

Su *et al*.^6^ demonstrated decreased bone mass in the metaphysis of the distal femur in *Fgfr3* ^G369C/+^ mice exhibiting gain-of-function in FGFR3 due to the elevated both osteoblastgenesis and osteoclastgenesis. Actually, bone mineral density was decreased in patients with human ACH^5^. The current study confirmed decreased trabecular bone in *Fgfr3* ^ach^ mice by radiological and histological analyses. We demonstrated that meclozine treatment increased bone mineral density of the metaphysis in *Fgfr3* ^ach^ mice. In addition to growth promotion effects in long tubular bones and vertebrae, improvement of the trabecular bone quality is another evidence that meclozine suppressed activated FGFR3 signalings in *in-vivo*.

Medical treatment for foramen magnum stenosis has a great clinical significance especially in young ACH children. In the current study, 1 mg/kg/day of meclozine increased the area of foramen magnum in *Fgfr3* ^ach^ mice, while 2 mg/kg/day did not show positive effects on foramen magnum area. Based on these conflicting results, we could not conclude the effect of meclozine on foramen magnum stenosis. Daily subcutaneous injection of BMN-111 could not increase the sagittal and lateral diameters of the foramen magnum in 7-day-old *Fgfr3* ^Y367C/+^ mice^13^. On the other hand, subcutaneous administration of NVP-BGJ398 from neonatal period increased the area of foramen magnum in *Fgfr3* ^Y367C/+^ mice^11^. We demonstrated that premature synchondroses closure around foramen magnum was already observed at day 4.5 in *Fgfr3* ^ach^ mice^20^. Timing of administration of FGFR3 inhibitors, therefore, is critical for the treatment of foramen magnum stenosis. Since oral administration was technically difficult for newborn mice, we could not rescue foramen magnum stenosis by meclozine treatment in the current study. Additionally, we have previously demonstrated that maternal *ad libitum* administration of meclozine also could not rescue foramen magnum stenosis in *Fgfr3* ^ach^ mice probably due to the low placental transmission of the drug^20^. The medical treatment for foramen magnum stenosis could be challenging even if effective FGFR3 inhibitors will be developed in the future.

Subcutaneous administration of NVP-BGJ398 was effective in other mouse models with short stature, such as hypophosphatemic rickets^21^ by reducing FGFR3 activation. In our previous study, *ad libitum* administration of meclozine promoted the body length in wild-type mice^16^. These results indicated that FGFR3 inhibitors might be applicable for short stature patients with other etiologies. Furthermore, oral administration (meclozine) is more convenient than injection (NVP-BGJ398 and BMN-111) in terms of long-term administration to young children.

There are several limitations in the current study. First, we employed *Fgfr3* ^ach^ mice among several mouse models of ACH. The body length of wild-type mice was longer by 4.5% than that of *Fgfr3* ^ach^ mice at the age of 7 days in the current study, indicating that the skeletal phenotype of *Fgfr3* ^ach^ mice seemed to be mild. Second, we could not determine the detailed *in-vivo* mechanisms of meclozine for promoting bone growth in *Fgfr3* ^ach^ mice, although we previously demonstrated inhibitory effects of meclozine on elevated FGFR3 signaling in chondrocytes^15^.

In conclusion, oral administration of clinically feasible dose of meclozine increased longitudinal bone growth in mouse model of ACH. In addition to short stature, meclozine ameliorated other skeletal phenotypes caused by excessive activity of FGFR3, including metaphyseal osteopenic conditions and relative thin cortical bones. The current *in-vivo* study indicated that meclozine had an inhibitory effect on abnormally activated FGFR3 signaling.

## Materials and Methods

### Ethics statement

Animal care and all experiments were conducted in accordance with the institutional guidelines of Nagoya University Graduate School of Medicine. All experimental protocols were approved by the institutional committee of Nagoya University Graduate School of Medicine.

### Mice

*Fgfr3* ^ach^ mice (FVB background) were provided by Dr. David M. Ornitz at Washington University^22^. In brief, *Fgfr3* ^ach^ mice express activated *FGFR3* in the growth plate using the *Col2a1* promoter. In all experiments, we used transgenic mice carrying the heterozygous *Fgfr3* ^ach^ transgene. Due to unavailability of a sufficient number of wild-type FVB mice, we employed ICR mice to investigate the plasma concentration of meclozine after a single dosage administration.

### Quantitative oral administration of meclozine

The 1, 2, or 20 mg/kg/day of meclozine (MP Biomedicals), which were prepared by mixing 0.1, 0.2, or 2 mg of meclozine with 1 ml of 0.5% methylcellulose (MC) (4000 centipoise; Sigma), were administered using feeding tube once or twice a day from 7 days of age for 10 days to *Fgfr3* ^ach^ mice which were born between *Fgfr3* ^ach^ male and wild-type female. On the other hand, 10 ml/kg/day of 0.5% MC was administrated to untreated mice. We genotyped mice using fingers by the age of 7 days. There were 93 *Fgfr3* ^ach^ mice and 55 wild-type mice in 34 kinships at the beginning of experiment at the age of 7 days. All of the wild-type littermates were untreated while *Fgfr3* ^ach^ mice were randomly treated with meclozine. Thus, three groups were generated in one kinship: 1) untreated wild-type mice, 2) untreated *Fgfr3* ^ach^ mice, 3) meclozine-treated *Fgfr3* ^ach^ mice. Among 93 *Fgfr3* ^ach^ mice, 31 *Fgfr3* ^ach^ mice with or without meclozine treatment were excluded because they had a significant paralysis in the lower extremities or died during the examination periods (Supplementary Fig. S6). For adjusting an environmental difference such as a litter size among kinships, the absolute values of the body length and radiological and histological bone parameters were compared within a kinship but not compared between kinships. We compared relative body lengths for untreated *Fgfr3* ^ach^ mice of the same-sex littermates before treatment (at the age of 7 days) among treatment groups. The mice that lacked a pair of untreated and meclozine-treated *Fgfr3* ^ach^ mice within the same kinships were also excluded. We finally analyzed a total of 19 kinships, including 54 *Fgfr3* ^ach^ mice and 34 wild-type mice.

### Radiological analysis

At the end of the treatment, the individual mice were subjected to a soft x-ray (30 kV, 5 mA for 20 s; SOFTEX Type CMB-2; SOFTEX) and micro-computed tomography (micro-CT) scans (0.5 mm Al filter, 50 kV, 500 μA for 0.054 seconds; SkyScan 1176, Bruker). Three-dimensional (3D) images of the whole body from the micro-CT scan were reconstructed by an in-house volume-rendering software^23^. This software enabled us to render 3D views of the micro-CT scan from arbitrary viewpoints and view directions as well as to measure the distance between two specific points, the specific area, and the specific bone volume. Using this software, we measured individual bone lengths, including the cranium, humerus, radius, ulna, femur, tibia, and vertebrae (L1–5), on the reconstructed 3D images. The area of foramen magnum, bone volumes of the cranium, upper extremity, lower extremity, and vertebrae (L1-5), and total bone volume were also calculated.

To evaluate the trabecular bone in the metaphysis of the distal femur, the 3D images reconstructed by the Skyscan NRecon software were analyzed using 3D algorithms in Skyscan CTAn software according to the manufacturer’s instructions. The 800 µm section starting below the growth plate was selected as metaphyseal trabecular region of interest (ROI). Bone volume/total volume (BV/TV), trabecular thickness (Tb.Th), trabecular number (Tb.N), and trabecular separation (Tb.Sp) were calculated as indices of metaphyseal trabecular compartments. For calculation of the bone mineral density (BMD) at the same sites of BV/TV measurements, calibration of the Skycan CT system was performed against a 0.25 g/cm^3^ and a 0.75 g/cm^3^ hydroxyapatite phantom.

### Histological analysis

The mice were transcardially perfused with 4% paraformaldehyde (PFA) under general anesthesia, and the right lower extremity was postfixed in 4% PFA at room temperature for 24 hours. After washing with phosphate buffered saline (PBS), the samples were decalcified in 10% EDTA solution at 4 °C for 3 weeks and then embedded in paraffin. Coronal thin sections (3 µm) were cut and stained with hematoxylin-eosin (HE). Images were captured with an Olympus BX53 microscope. The areas of metaphyseal trabecular bone stained with HE were measured by Image J software in blinded manner.

### Measurement of plasma concentrations of meclozine

We employed 8-week-old ICR mice to investigate the plasma concentration, because it is technically difficult to obtain sufficient amount of plasma from younger mice. Cardiac blood samples were collected under anesthesia at 0.25, 0.5, 1, 2, 4, 8, 12, or 24 hours after single dose of 2, 6, or 20 mg/kg of meclozine. The deproteinated plasma of meclozine adding with internal standard substance was subjected to Liquid Chromatography – tandem Mass Spectrometry. Inert Sustain C18 (GL Science Inc.) was used as an Ultra-Performance Liquid Chromatgraphy column. The gradient elution was performed by using water/formic acid (1000:1, v/v) and acetonitrile. Plasma concentrations of meclozine were measured in each sample (LSI Medience Co.). The plasma concentration-time data were analyzed by non-compartmental analysis (with the aid of WinNonlin software, version 6.3, Pharsight Corporation as part of Certara). The peak drug concentration (C_max_) and time to peak drug concentration (T_max_) were obtained directly from the original concentration-time data. The terminal elimination half-life (T_1/2_) was automatically calculated by WinNonlin software using concentrations at 3 to 6 time points in the terminal phase. The area under the concentration-time curve from time zero to the last sampling time 24 hours (AUC_0-24_) and area under the concentration-time curve from time zero to infinity (AUC_inf_) were also automatically calculated by WinNonlin software. Pharmacokinetics after repeated dose was simulated by means of the individual pharmacokinetic parameters. Concentration-time curves were simulated on 2, 6, or 20 mg/kg of meclozine every 12 hours for 7 days (LSI Medience Co.).

### Statistical analysis

Data are expressed as mean ± SD. Statistical analyses were carried out using the unpaired Student *t* test.

## Electronic supplementary material

Supplementary Information
