# Supplementary material for: Clinical dosage of meclozine promotes longitudinal bone growth, bone volume, and trabecular bone quality in transgenic mice with achondroplasia
Source: Sci Rep. 2017 Aug 7;7:7371. doi: 10.1038/s41598-017-07044-8 (PMC5547068; doi:10.1038/s41598-017-07044-8)

Clinical dosage of meclozine promotes longitudinal bone growth, bone volume, and trabecular bone quality in transgenic mice with achondroplasia

Masaki Matsushita,[1,2] Ryusaku Esaki,[1,2] Kenichi Mishima,[1,2] Naoki Ishiguro,[1] Kinji Ohno,[2] and Hiroshi Kitoh [1]

[1]Department of Orthopaedic Surgery, Nagoya University Graduate School of Medicine, 65 Tsurumai, Showa-ku, Nagoya 466-8550, Japan. [2]Division of Neurogenetics, Center for Neurological Diseases and Cancer, Nagoya University Graduate School of Medicine, 65 Tsurumai, Showa-ku, Nagoya 466-8550, Japan. Correspondence and requests for materials should be addressed to H.K. (email: hkitoh@med.nagoya-u.ac.jp)

Supplementary Figure S1. Cumulative pharmacokinetics is observed after repeated administration of 20 mg/kg of meclozine for 7 days. (**A**) Plasma concentration-time course after the first administration of indicated doses was shown. (**B**) Simulated curved after the 14th administration of indicated doses was shown. (**C**) Panel A and B were merged. ‘q 12 h’ means every 12 hours.

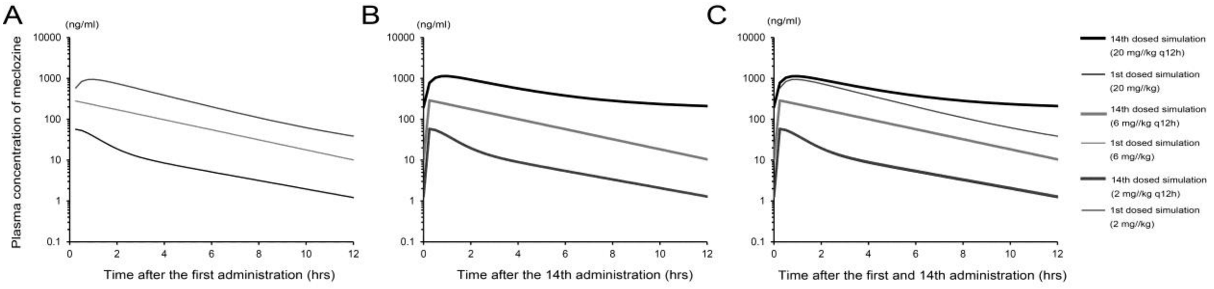

Supplementary Figure S2. The *Fgfr3*^{ach} mouse treated with twice-daily administration of 1 mg/kg/day of meclozine is slightly larger than untreated *Fgfr3*^{ach} mouse. The photo (**A**) and 3D image reconstructed from micro-CT scan (**B**) show the same-sex littermates of *Fgfr3*^{ach} mice treated with or without twice-daily administration of 1 mg/kg/day of meclozine.

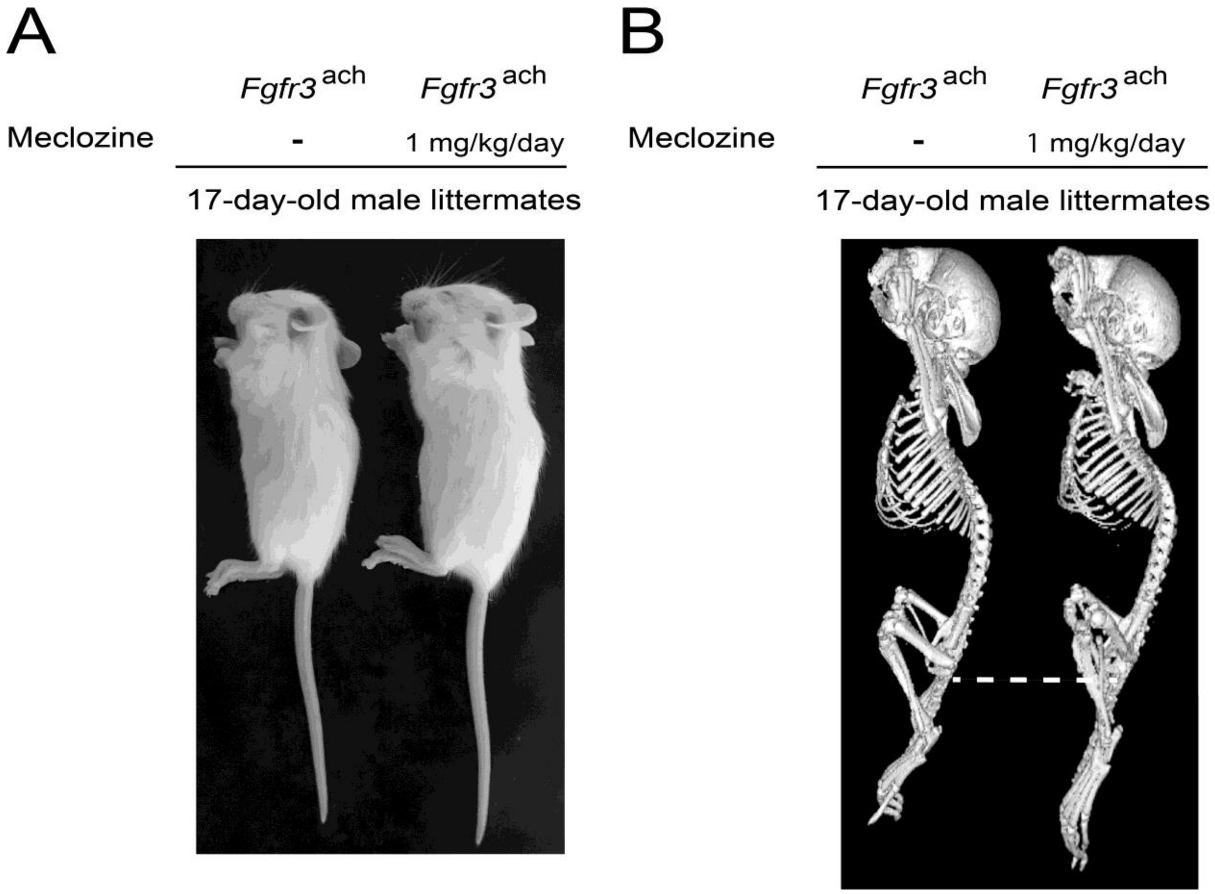

Supplementary Figure S3. The *Fgfr3*^{ach} mouse treated with twice-daily administration of 20 mg/kg/day of meclozine is not larger than untreated *Fgfr3*^{ach} mouse. The photo (**A**) and 3D image reconstructed from micro-CT scan (**B**) show the same-sex littermates of *Fgfr3*^{ach} mice treated with or without twice-daily administration of 20 mg/kg/day of meclozine.

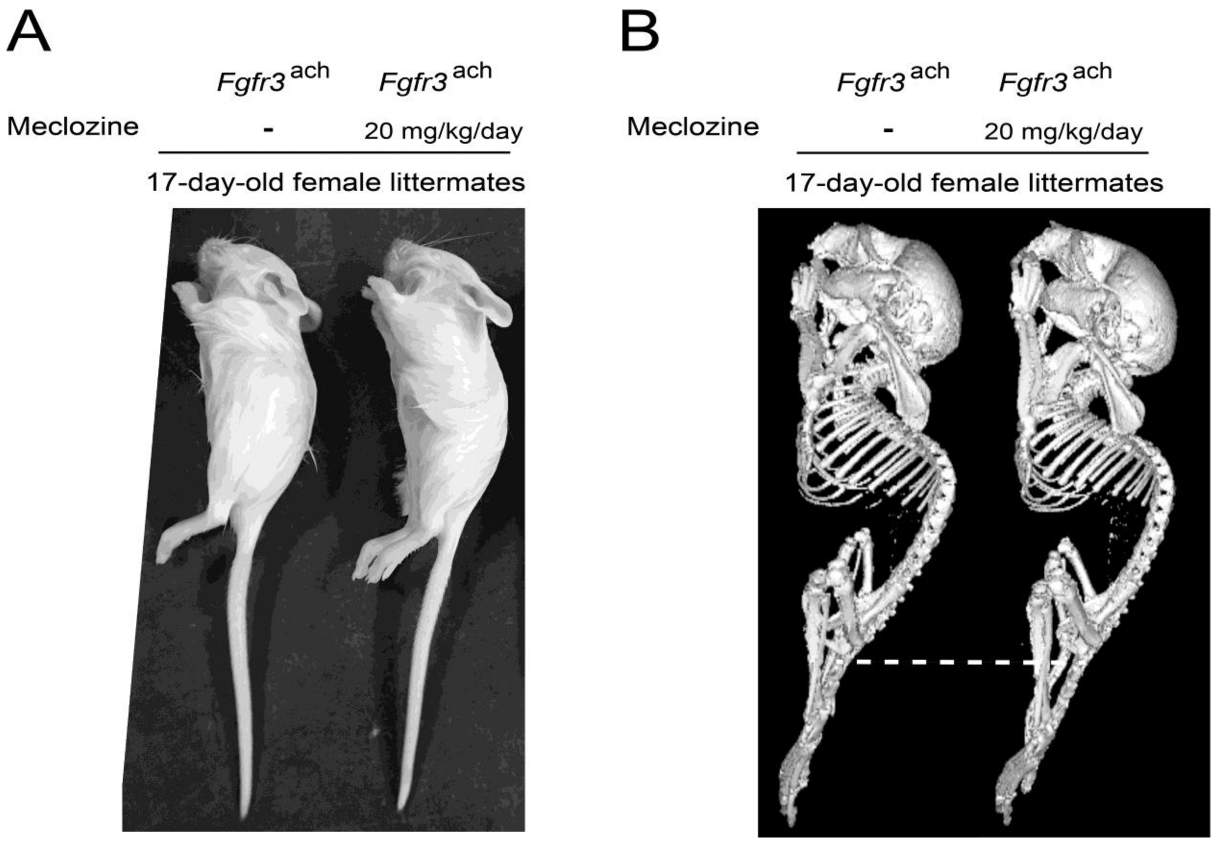

Supplementary Figure S4. Once-daily administration of 1 and 2 mg/kg/day of meclozine does not increase the body length in *Fgfr3*^{ach} mice. Relative body length of the *Fgfr3*^{ach} mice did not change after once-daily administration of meclozine for 10 days. Mean and SD are indicated. Statistical significance was analyzed by the unpaired *t* test for each dose of meclozine-treated *Fgfr3*^{ach} mice (n = 8 and 5 for 1 and 2 mg/kg/day of meclozine, respectively) or untreated wild-type (n = 18) mice versus untreated *Fgfr3*^{ach} mice (n = 10).

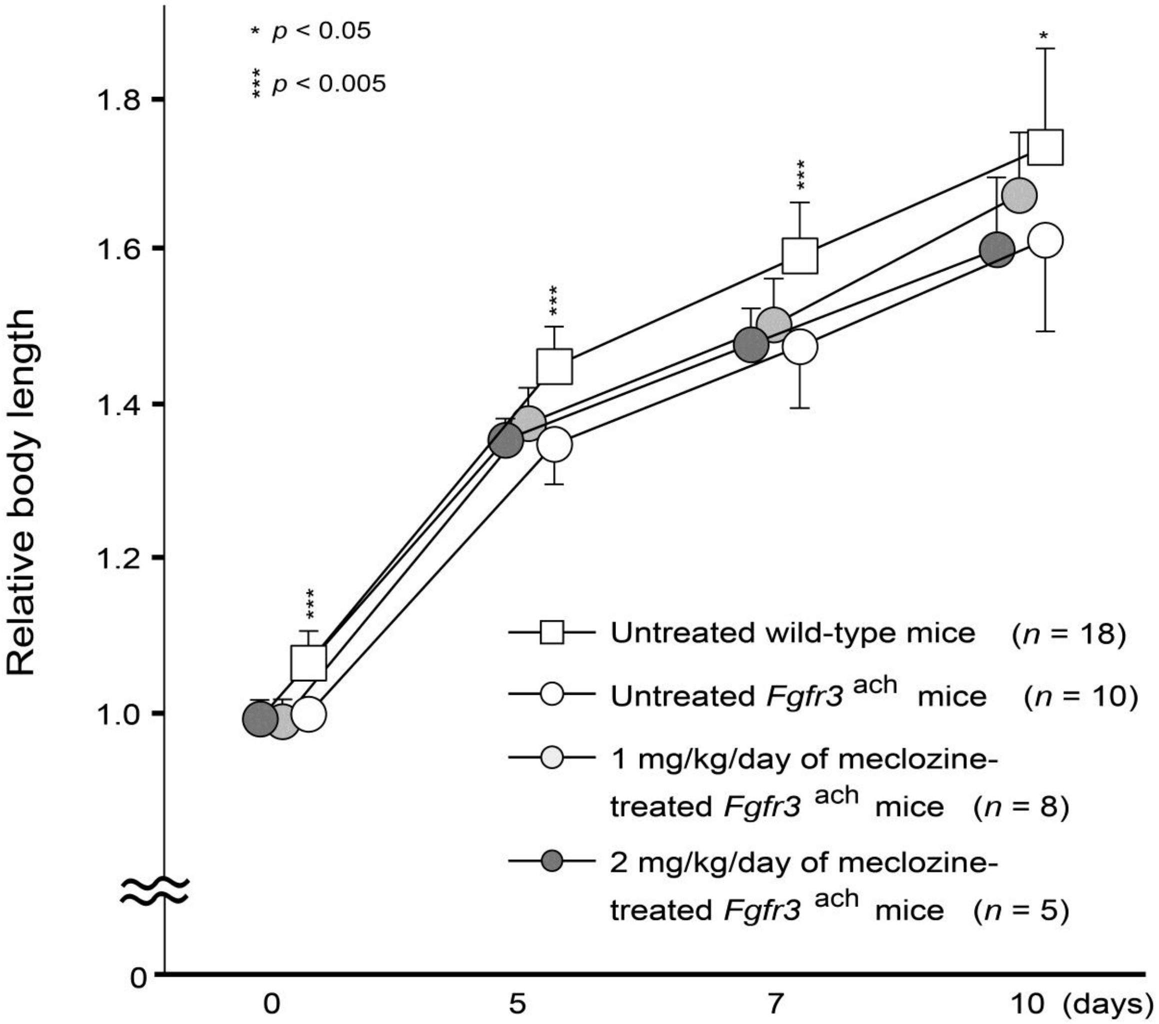

Supplementary Figure S5. The histology images show the trabecular bone in the metaphysis of *Fgfr3*^{ach} mice with or without 1 mg/kg/day of meclozine treatment.

Representative decalcified histology images of the distal femur stained with hematoxylin-eosin (HE) demonstrated that the trabecular bone was more abundant in 1 mg/kg/day of meclozine-treated *Fgfr3*^{ach} mouse than in untreated *Fgfr3*^{ach} mouse. Squared parts are magnified in lower panels. Scale bares indicate 400 μm in upper panels and 100 μm in lower panels.

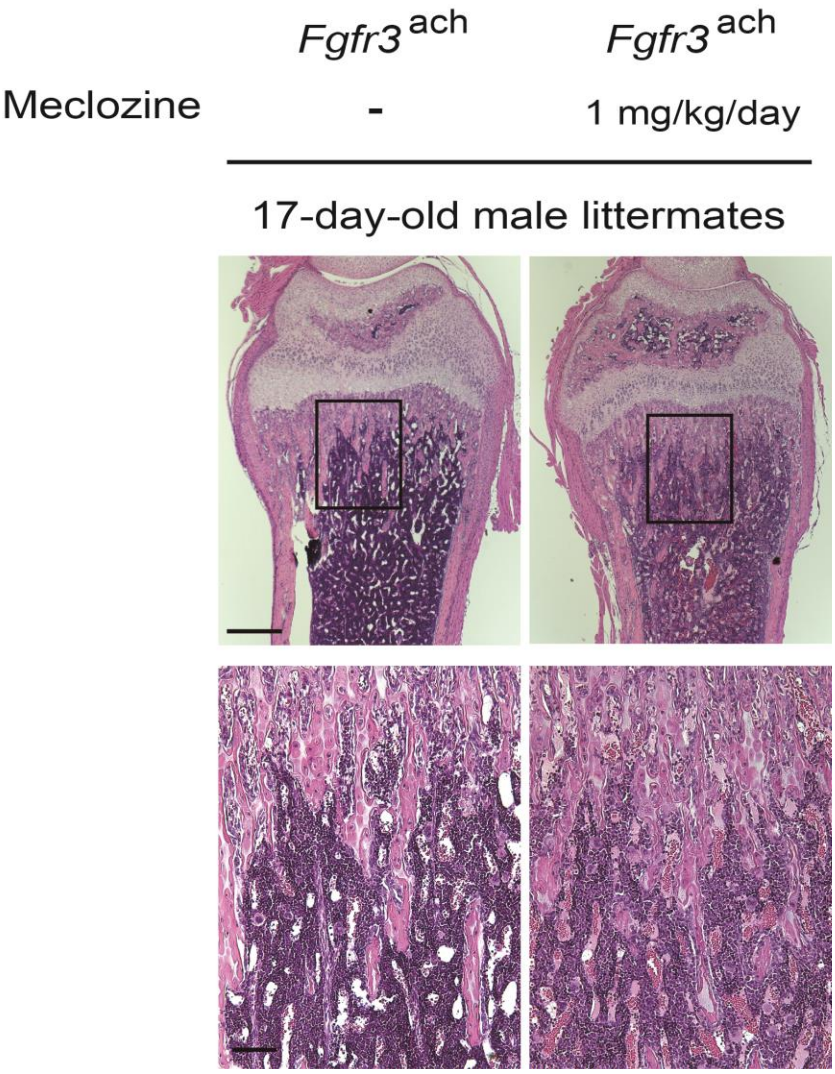

Supplementary Figure S6. Kaplan-Meier curve shows the rate of mice that had neither lower extremity paralysis nor mortality in *Fgfr3*^{ach} mice. There were no favorable effects of meclozine on the rate of complications or mortality with *Fgfr3*^{ach} mice.

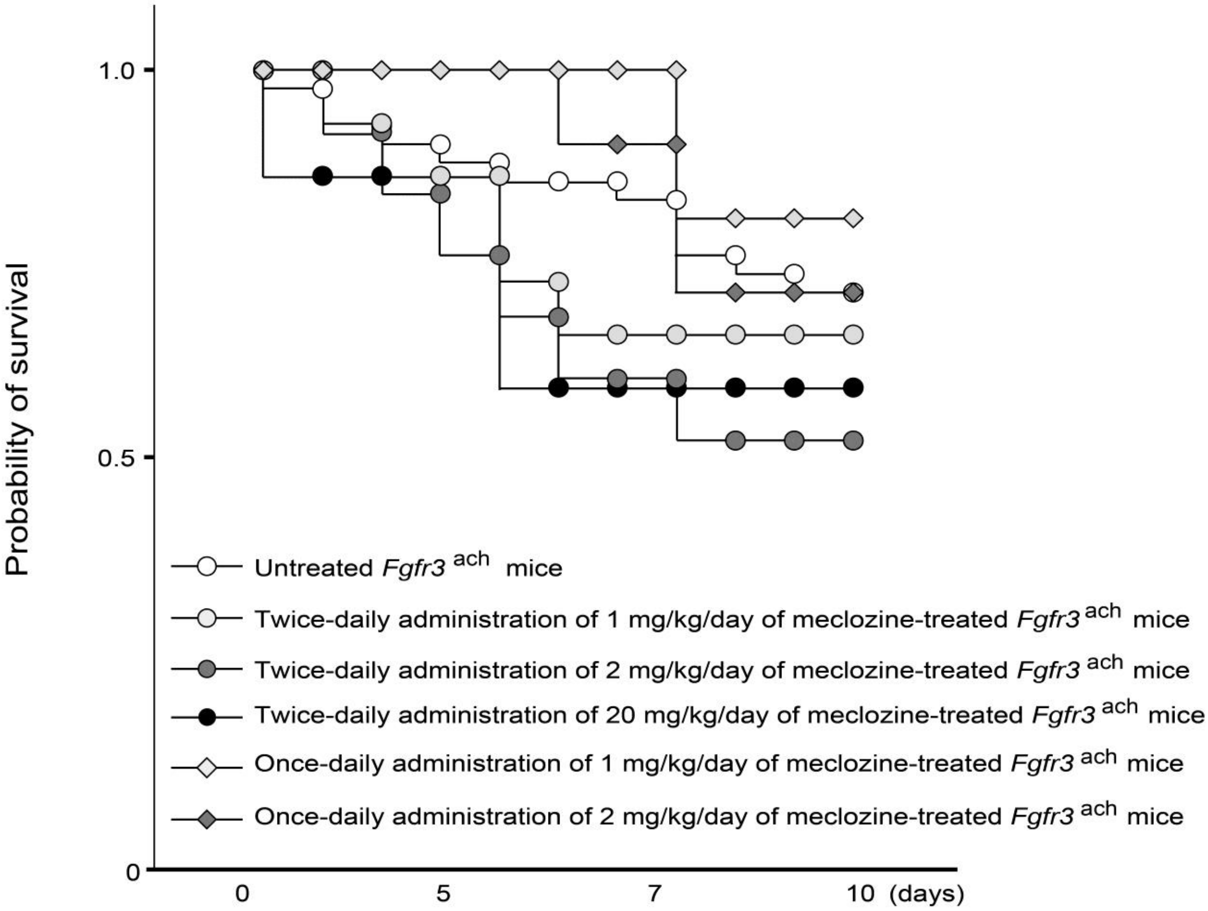

Supplement: Supplementary file 1 — Supplementary Information [file 41598_2017_7044_MOESM1_ESM.pdf]
